# Isonicotinium hydrogen sulfate

**DOI:** 10.1107/S1600536809034916

**Published:** 2009-09-05

**Authors:** Li-Zhuang Chen

**Affiliations:** a Ordered Matter Science Research Center, College of Chemistry and Chemical Engineering, Southeast University, Nanjing 210096, People’s Republic of China

## Abstract

The crystal structure of the title compound, C_6_H_6_NO_2_^+^·HSO_4_^−^, is stabilized by inter­molecular N—H⋯O and O—H⋯O hydrogen bonds.

## Related literature

For background to simple mol­ecular–ionic crystals containing organic cations and acidic anions (1:1 molar ratio), see: Czupiński *et al.* (2002 ▶); Katrusiak & Szafrański (1999 ▶, 2006 ▶). For a related structure, see: Jebas *et al.* (2006 ▶).
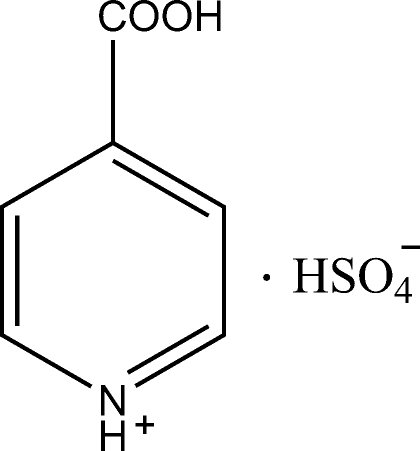


## Experimental

### 

#### Crystal data

C_6_H_6_NO_2_^+^·HSO_4_^−^
*M* *_r_* = 221.18
Monoclinic, 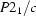

*a* = 8.3816 (17) Å
*b* = 11.439 (2) Å
*c* = 9.4057 (19) Å
β = 109.12 (3)°
*V* = 852.0 (3) Å^3^
*Z* = 4
Mo *K*α radiation
μ = 0.39 mm^−1^
*T* = 293 K
0.25 × 0.22 × 0.20 mm

#### Data collection

Rigaku SCXmini diffractometer
Absorption correction: multi-scan (*CrystalClear*; Rigaku, 2005 ▶) *T*_min_ = 0.90, *T*_max_ = 0.92
8697 measured reflections
1947 independent reflections
1745 reflections with *I* > 2σ(*I*)
*R*_int_ = 0.043

#### Refinement

*R*[*F* ^2^ > 2σ(*F* ^2^)] = 0.036
*wR*(*F* ^2^) = 0.091
*S* = 1.14
1947 reflections
128 parameters
H-atom parameters constrained
Δρ_max_ = 0.29 e Å^−3^
Δρ_min_ = −0.38 e Å^−3^

### 

Data collection: *CrystalClear* (Rigaku, 2005 ▶); cell refinement: *CrystalClear*; data reduction: *CrystalClear*; program(s) used to solve structure: *SHELXS97* (Sheldrick, 2008 ▶); program(s) used to refine structure: *SHELXL97* (Sheldrick, 2008 ▶); molecular graphics: *SHELXTL* (Sheldrick, 2008 ▶); software used to prepare material for publication: *SHELXL97*.

## Supplementary Material

Crystal structure: contains datablocks I, global. DOI: 10.1107/S1600536809034916/pv2195sup1.cif

Structure factors: contains datablocks I. DOI: 10.1107/S1600536809034916/pv2195Isup2.hkl

Additional supplementary materials:  crystallographic information; 3D view; checkCIF report

## Figures and Tables

**Table 1:** Hydrogen-bond geometry (Å, °)

*D*—H⋯*A*	*D*—H	H⋯*A*	*D*⋯*A*	*D*—H⋯*A*
O2—H2*B*⋯O6^i^	0.85	1.81	2.6425 (19)	166
O3—H3⋯O5^ii^	0.94	1.75	2.6543 (18)	160
N1—H1*A*⋯O5	0.86	1.94	2.787 (2)	167

Symmetry codes: (i) 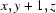; (ii) 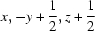.

**Fig. 1.**
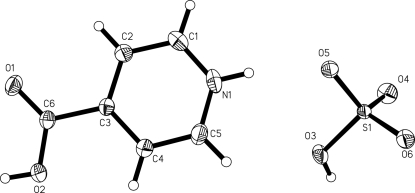
The asymmetric unit of the title compound with atom labels; the thermal elliposids have been drawn at 50% probability level.

**Fig. 2.**
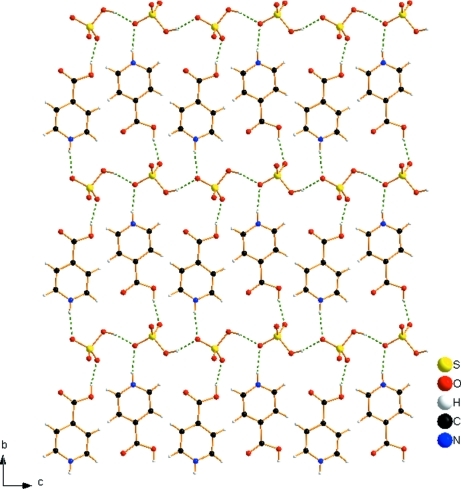
The H-bonded two-dimensional network of the title compound viewed down the a axis. Hydrogen bonds are drawn as dashed lines

## supplementary crystallographic information

### Comment

Recently, much attention has been devoted to simple molecular–ionic crystals containing organic cations and acid radicals (1:1molar ratio) due to the tunability of their special structural features and their interesting physical properties (Czupiński *et al.*, 2002; Katrusiak & Szafrański, 1999; Katrusiak & Szafrański, 2006). The crystal structure of isonicotinium nitrate monohydrate has been reported. (Jebas *et al.*, 2006). In our laboratory, the title compound, (I), has been synthesized, its crystal structure is reported herein.

The asymmetric unit of the title compound consists of protoned isonicotinic acid  C_6_H_6_NO_2_^+^ and HSO_4_^-^anions (Fig. 1). The isonicotinium cation is essentially planar. The crystal structure is stabilized by intermolecular N—H···O and O—H···O hydrogen bonds. The H-bonds form a two-dimensional network as presented in Fig 2. viewed along the *a*-axis.

### Experimental

Isonicotinic acid (10 mmol) and 10% aqueous H_2_SO_4_ in a molar ratio of 1:1 were mixed and dissolved in water by heating to 353 K forming a clear solution. The reaction mixture was cooled slowly to room temperature, crystals of the title compound were formed, collected and washed with dilute aqueous H_2_SO_4_.

### Refinement

Hydrogen atoms bonded to N and O were located from a difference map and were included at those positions (O—H = 0.85/0.95 Å and N—H = 0.86 Å), while the remaining H atoms were placed in calculated positions, with C—H = 0.93 Å, and refined using a riding model, with *U*_iso_(H)=1.2*U*_eq_(C,*N*) and 1.5*U*_eq_(O).

### Crystal data

**Table:**

C_6_H_6_NO_2_^+^·HSO_4_^−^	*F*(000) = 456
*M**_r_* = 221.18	*D*_x_ = 1.716 Mg m^−^^3^
Monoclinic, *P*2_1_/*c*	Mo *K*α radiation, λ = 0.71073 Å
Hall symbol: -P 2ybc	Cell parameters from 1745 reflections
*a* = 8.3816 (17) Å	θ = 3.1–27.5°
*b* = 11.439 (2) Å	µ = 0.39 mm^−^^1^
*c* = 9.4057 (19) Å	*T* = 293 K
β = 109.12 (3)°	Block, colorless
*V* = 852.0 (3) Å^3^	0.25 × 0.22 × 0.2 mm
*Z* = 4	

### Data collection

**Table:**

Rigaku SCXmini diffractometer	1947 independent reflections
Radiation source: fine-focus sealed tube	1745 reflections with *I* > 2σ(*I*)
graphite	*R*_int_ = 0.043
Detector resolution: 13.6612 pixels mm^-1^	θ_max_ = 27.5°, θ_min_ = 3.1°
ω scans	*h* = −10→10
Absorption correction: multi-scan (*CrystalClear*; Rigaku, 2005)	*k* = −14→14
*T*_min_ = 0.90, *T*_max_ = 0.92	*l* = −12→12
8697 measured reflections	

### Refinement

**Table:**

Refinement on *F*^2^	Secondary atom site location: difference Fourier map
Least-squares matrix: full	Hydrogen site location: inferred from neighbouring sites
*R*[*F*^2^ > 2σ(*F*^2^)] = 0.036	H-atom parameters constrained
*wR*(*F*^2^) = 0.091	*w* = 1/[σ^2^(*F*_o_^2^) + (0.0324*P*)^2^ + 0.395*P*] where *P* = (*F*_o_^2^ + 2*F*_c_^2^)/3
*S* = 1.14	(Δ/σ)_max_ < 0.001
1947 reflections	Δρ_max_ = 0.29 e Å^−^^3^
128 parameters	Δρ_min_ = −0.38 e Å^−^^3^
0 restraints	Extinction correction: *SHELXL97* (Sheldrick, 2008), Fc^*^=kFc[1+0.001xFc^2^λ^3^/sin(2θ)]^-1/4^
Primary atom site location: structure-invariant direct methods	Extinction coefficient: 0.164 (6)

### Special details

**Table:**

Geometry. All e.s.d.'s (except the e.s.d. in the dihedral angle between two l.s. planes) are estimated using the full covariance matrix. The cell e.s.d.'s are taken into account individually in the estimation of e.s.d.'s in distances, angles and torsion angles; correlations between e.s.d.'s in cell parameters are only used when they are defined by crystal symmetry. An approximate (isotropic) treatment of cell e.s.d.'s is used for estimating e.s.d.'s involving l.s. planes.
Refinement. Refinement of *F*^2^ against ALL reflections. The weighted *R*-factor *wR* and goodness of fit *S* are based on *F*^2^, conventional *R*-factors *R* are based on *F*, with *F* set to zero for negative *F*^2^. The threshold expression of *F*^2^ > σ(*F*^2^) is used only for calculating *R*-factors(gt) *etc*. and is not relevant to the choice of reflections for refinement. *R*-factors based on *F*^2^ are statistically about twice as large as those based on *F*, and *R*- factors based on ALL data will be even larger.

### Fractional atomic coordinates and isotropic or equivalent isotropic displacement parameters (Å^2^)

**Table:**

	*x*	*y*	*z*	*U*_iso_*/*U*_eq_	
S1	0.73722 (6)	0.21100 (4)	0.56236 (4)	0.02476 (17)	
O2	0.86500 (19)	0.91218 (12)	0.57234 (16)	0.0404 (4)	
H2B	0.8723	0.9863	0.5727	0.061*	
O6	0.88648 (17)	0.14017 (12)	0.62022 (16)	0.0392 (4)	
O1	0.71828 (19)	0.93897 (12)	0.32951 (16)	0.0405 (4)	
O5	0.72948 (19)	0.26942 (12)	0.42296 (14)	0.0372 (4)	
O4	0.58610 (18)	0.15100 (13)	0.55516 (17)	0.0427 (4)	
O3	0.7598 (2)	0.31698 (11)	0.67114 (15)	0.0413 (4)	
H3	0.7346	0.3019	0.7594	0.062*	
C6	0.7789 (2)	0.87673 (16)	0.4359 (2)	0.0293 (4)	
C3	0.7616 (2)	0.74617 (16)	0.42727 (19)	0.0270 (4)	
C4	0.8443 (3)	0.67611 (17)	0.5487 (2)	0.0354 (4)	
H4A	0.9152	0.7089	0.6374	0.042*	
C2	0.6584 (2)	0.69616 (17)	0.2959 (2)	0.0346 (4)	
H2A	0.6026	0.7427	0.2135	0.041*	
N1	0.7200 (2)	0.51277 (15)	0.4078 (2)	0.0423 (4)	
H1A	0.7067	0.4382	0.4019	0.051*	
C5	0.8204 (3)	0.55833 (18)	0.5365 (2)	0.0418 (5)	
H5A	0.8739	0.5099	0.6175	0.050*	
C1	0.6393 (3)	0.57727 (19)	0.2881 (2)	0.0409 (5)	
H1B	0.5707	0.5420	0.2002	0.049*	

### Atomic displacement parameters (Å^2^)

**Table:**

	*U*^11^	*U*^22^	*U*^33^	*U*^12^	*U*^13^	*U*^23^
S1	0.0360 (3)	0.0205 (2)	0.0191 (2)	−0.00064 (16)	0.01072 (17)	−0.00045 (14)
O2	0.0547 (9)	0.0241 (7)	0.0376 (8)	−0.0021 (6)	0.0087 (6)	−0.0019 (6)
O6	0.0381 (8)	0.0309 (7)	0.0407 (8)	0.0034 (6)	0.0023 (6)	−0.0038 (6)
O1	0.0506 (9)	0.0312 (7)	0.0398 (8)	0.0061 (6)	0.0148 (7)	0.0096 (6)
O5	0.0644 (10)	0.0292 (7)	0.0217 (7)	−0.0011 (6)	0.0194 (6)	−0.0001 (5)
O4	0.0385 (8)	0.0453 (9)	0.0477 (9)	−0.0067 (6)	0.0185 (6)	0.0008 (7)
O3	0.0794 (11)	0.0246 (7)	0.0280 (7)	−0.0030 (7)	0.0285 (7)	−0.0051 (5)
C6	0.0293 (9)	0.0258 (9)	0.0345 (10)	0.0018 (7)	0.0128 (7)	0.0022 (7)
C3	0.0284 (9)	0.0258 (9)	0.0294 (9)	0.0011 (7)	0.0130 (7)	0.0014 (7)
C4	0.0413 (11)	0.0296 (9)	0.0332 (10)	0.0013 (8)	0.0094 (8)	0.0017 (8)
C2	0.0341 (10)	0.0337 (10)	0.0343 (10)	0.0003 (8)	0.0090 (8)	0.0002 (8)
N1	0.0540 (11)	0.0234 (8)	0.0569 (12)	−0.0060 (7)	0.0282 (9)	−0.0041 (7)
C5	0.0550 (13)	0.0302 (10)	0.0412 (12)	0.0032 (9)	0.0171 (10)	0.0065 (9)
C1	0.0397 (11)	0.0389 (11)	0.0437 (12)	−0.0079 (9)	0.0133 (9)	−0.0110 (9)

### Geometric parameters (Å, °)

**Table:**

S1—O4	1.4226 (15)	C3—C4	1.382 (3)
S1—O6	1.4393 (14)	C4—C5	1.361 (3)
S1—O5	1.4540 (13)	C4—H4A	0.9300
S1—O3	1.5574 (13)	C2—C1	1.369 (3)
O2—C6	1.314 (2)	C2—H2A	0.9300
O2—H2B	0.8500	N1—C1	1.331 (3)
O1—C6	1.197 (2)	N1—C5	1.334 (3)
O3—H3	0.9372	N1—H1A	0.8600
C6—C3	1.500 (3)	C5—H5A	0.9300
C3—C2	1.379 (3)	C1—H1B	0.9300
			
O4—S1—O6	113.36 (9)	C5—C4—H4A	120.5
O4—S1—O5	113.76 (9)	C3—C4—H4A	120.5
O6—S1—O5	112.10 (9)	C1—C2—C3	119.23 (19)
O4—S1—O3	108.75 (9)	C1—C2—H2A	120.4
O6—S1—O3	106.64 (9)	C3—C2—H2A	120.4
O5—S1—O3	101.22 (8)	C1—N1—C5	123.11 (18)
C6—O2—H2B	109.1	C1—N1—H1A	118.4
S1—O3—H3	115.1	C5—N1—H1A	118.4
O1—C6—O2	125.44 (18)	N1—C5—C4	119.67 (19)
O1—C6—C3	122.68 (17)	N1—C5—H5A	120.2
O2—C6—C3	111.87 (15)	C4—C5—H5A	120.2
C2—C3—C4	119.86 (18)	N1—C1—C2	119.18 (19)
C2—C3—C6	118.85 (16)	N1—C1—H1B	120.4
C4—C3—C6	121.28 (16)	C2—C1—H1B	120.4
C5—C4—C3	118.95 (19)		

### Hydrogen-bond geometry (Å, °)

**Table:**

*D*—H···*A*	*D*—H	H···*A*	*D*···*A*	*D*—H···*A*
O2—H2B···O6^i^	0.85	1.81	2.6425 (19)	166
O3—H3···O5^ii^	0.94	1.75	2.6543 (18)	160
N1—H1A···O5	0.86	1.94	2.787 (2)	167

Symmetry codes: (i) *x*, *y*+1, *z*; (ii) *x*, −*y*+1/2, *z*+1/2.
